# AI-Enabled Customer Relationship Management Platforms for Patient Services in Health Care, Early Lessons From Governance, and Program-Level Outcomes

**DOI:** 10.2196/83564

**Published:** 2026-02-02

**Authors:** Anup Kant Gupta

**Affiliations:** 1 Strategic Management & Leadership Kelley School of Business Indiana University Bloomington Bloomington, IN United States

**Keywords:** artificial intelligence, health care customer relationship management, salesforce, patient affordability, patient adherence, patient access, digital transformation, governance

## Abstract

This research letter summarizes early lessons from 4 enterprise implementations of artificial intelligence–enabled customer relationship management platforms in health care and describes governance practices associated with improvements in affordability, adherence, and access at program level.

## Introduction

Health systems continue to face pressure to improve patient outcomes while safeguarding affordability and equitable access [1-4]. Electronic health records support clinical workflows but are not designed for proactive patient outreach or service coordination [5]. Artificial intelligence (AI)-enabled customer relationship management (CRM) platforms support automated eligibility checks, outreach, and risk alerts [6-8]. This research letter summarizes early descriptive insights from enterprise implementations of AI-enabled CRM platforms.

## Methods

### Program Selection and Sampling Frame

A case-informed thematic analysis was conducted across 4 large health care programs. These were the largest CRM-based patient service deployments accessible to the author during this period. Programs were included if they had live AI-enabled CRM workflows for affordability, adherence, or access for at least 12 months. Each program operated independently with different teams, geographies, and product lines. Data reflect operations recorded between January 2019 and March 2024 across the 4 programs. All outcomes reflect a descriptive analysis of the operational dashboards and do not infer causal relationships.

### Data Sources and Definitions

The following three data sources were used, each aggregated at program level.

1. Governance documents summarizing decision logs, compliance checkpoints, and escalation patterns.
2. Stakeholder feedback was gathered through routine program review meetings and documented in standard internal templates.
3. Internal dashboards tracking key operational indicators. These dashboards were deidentified, contained no patient level data, and were part of routine program monitoring.

Program level outcome metrics were defined as follows.

1. Adoption: proportion of active CRM users among provisioned patient service staff within a measurement window.
2. Affordability efficiency: average time for co-pay or financial assistance verification.
3. Therapy initiation time: average days between benefit verification and therapy start.
4. Discontinuation rate: proportion of enrolled patients who stopped therapy during a defined period.

Baseline values came from preimplementation operations, and follow-up reflected the first stable post launch period. Findings are observational.

### Ethical Considerations

This work used only aggregated, deidentified operational dashboards and program documents. No patient-level identifiable data or human subject interaction occurred. Institutional review board approval was not required.

## Results

### Theme 1: Patient Outcome Alignment and Sponsorship

Programs that centered goals on patient experience and outcomes such as therapy initiation time or affordability enrollment sustained stronger executive sponsorship. Programs framed as IT upgrades struggled to maintain alignment.

### Theme 2: Continuous Engagement and Adoption

High adoption correlated with structured engagement of patient service teams and compliance officers. Regular feedback cycles supported adoption above 85% vs below 60% in minimally engaged programs [2].

### Theme 3: Hybrid Governance Improved Delivery Efficiency

**Figure 1:**
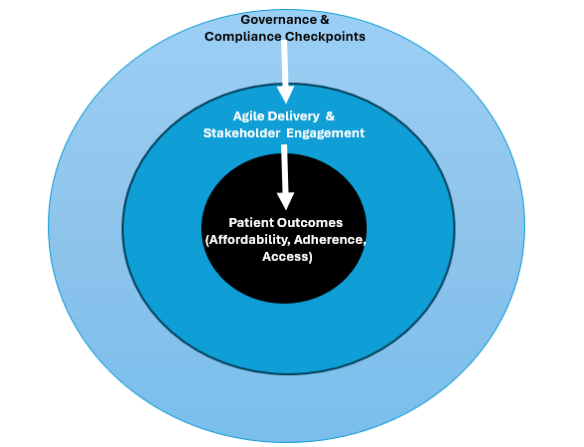
Hybrid governance framework for artificial intelligence–enabled customer relationship management. The framework illustrates three concentric layers: (1) patient outcomes (affordability, adherence, access) at the core, (2) agile delivery and stakeholder engagement in the middle layer, and (3) governance and compliance checkpoints in the outer layer. Arrows depict how governance structures enable engagement, which in turn drives improved patient outcomes.

All 4 programs used a hybrid model that combined Agile sprints with scheduled compliance reviews. This approach reduced backlog resolution time by about 30%. Figure 1 summarizes the governance structure.

### Theme 4: Program-Level Improvements in Affordability, Adherence, and Access

Changes reflect aggregated dashboards and are not causal. Examples include a reduction in co-pay verification time from 4 days to 3 days (25% faster), a 12% reduction in therapy discontinuation rates, and a shortening of therapy initiation time from 20 days to 17 days (15% faster) (Table 1).

**Table 1:** Program level customer relationship management–enabled improvements.

Patient service	Artificial intelligence functionality	Outcome	Example (anonymized)
Affordability	Automated co-pay verification	Reduced processing time (4 days to 3 days, a 25% improvement)	Fortune 10 rollout (USA)
Adherence	Predictive risk alerts	12% reduction in therapy discontinuation	Consulting-led program
Access	Automated prior authorization	Therapy initiation shortened (20 days to 17 days, a 15% improvement)	Enterprise-wide program

## Discussion

### Summary of Findings

Across 4 large-scale implementations, AI-enabled CRM platforms supported improvements in affordability, adherence, and access through workflow automation, risk identification, and coordinated service tasks. The most consistent predictors of success were early alignment with patient-centered outcomes, continuous stakeholder engagement, and hybrid governance structures.

### Interpretation and Limitations

Results reflect aggregated, unaudited operational dashboards and cannot establish causality [9,10]. Findings describe program-level improvements observed post implementation and may not generalize to organizations with different scales, regulatory environments, or infrastructure maturity.

### Future Directions

Future studies should incorporate multi-site comparative designs, validated outcomes, and integration with digital therapeutics and device streams to strengthen evidence for CRM-enabled patient service transformation.

## Abbreviations

AI: artificial intelligence
CRM: customer relationship management
